# Maintenance Cost Minimization for an Agricultural Harvesting Gripper

**DOI:** 10.3390/s23084103

**Published:** 2023-04-19

**Authors:** Florina Maria Șerdean, Mihai Dan Șerdean, Silviu-Dan Mândru

**Affiliations:** 1 Faculty of Industrial Engineering, Robotics and Management Production, Technical University of Cluj-Napoca, 103-105 Muncii Blvd., 400641 Cluj-Napoca, Romania; 2 Faculty of Automotive, Mechatronics and Mechanical Engineering, Technical University of Cluj-Napoca, 103-105 Muncii Blvd., 400641 Cluj-Napoca, Romania

**Keywords:** optimization, evolutionary algorithm, maintenance cost minimization, smart farming, fluidic muscles, preventive maintenance

## Abstract

A crucial aspect that has to be considered in all fields and, especially, in smart farming, a rapidly developing industry, is maintenance. Due to the costs generated by both under-maintaining and over-maintaining the components of a system, a balance has to be achieved. The paper is focused on presenting an optimal maintenance policy used to ensure cost minimization by determining the optimal time to make a preventive replacement of the actuators of a harvesting robotic system. First, a brief presentation of the gripper with Festo fluidic muscles used in a novel way instead of fingers is given. Then, the nature-inspired optimization algorithm, as well as the maintenance policy are described. The paper also includes the steps and the obtained results of the developed optimal maintenance policy applied for the Festo fluidic muscles. The outcome of the optimization shows that a significant reduction in the costs is obtained if one performs a preventive replacement of the actuators a few days before the lifetime provided by the manufacturer and the lifetime estimated using a Weibull distribution.

## 1. Introduction

In the last decades, a key role in increasing productivity and reducing costs in terms of industrial agricultural production has been played by robotics and automation. However, high implementation costs and failed results have slowed down the research activities in the field of robotics for agriculture. Recently, more projects and more useful applications have emerged in this field due to the rising cost of labor and new regulations on occupational safety.

Researchers are highly interested in mechanization and automatic harvesting as they can effectively reduce the overall costs of agricultural production. Nevertheless, due to the variations in fruit and legume sizes and shapes, as well as different harvesting techniques, researchers must develop customized robotic harvesting systems for different crop categories [1].

When designing independent robotic systems for harvesting crops such as fruits, several issues should be addressed. The main ones are enumerated in [2]: “high precision in fruit identification, real-time decision making about harvesting and storing, handling without deteriorating both the plant and the fruit”. Additionally, the cost of designing and maintaining such a robotic system must be considered because there are studies whose outcome indicates that to be successful on the market, the harvesting costs must be reduced by half [1].

Research regarding harvesting grippers shows that different manipulator structures were developed considering the crop, such as a gripper with soft tips fingers [3], soft protection [4], or a soft structure [5], a gripper with spoon-shaped gripping elements [6], a vacuum-grasping dual arm robot [7], and so on. An overview of the agricultural grippers used to control weeds and harvesting is presented in [1] in an easy-to-read graphical list. Another comprehensive overview in the form of a table summary of the techno-functional properties of the different types of agricultural grippers is encompassed in [2].

In the context of the accelerated development of technology, maintenance has become an increasingly topical subject due to the fact that when it is lacking, or when it is overlooked, the consequences can be tragic in most industries. This is also true for the smart farming industry. However, a balance must be kept between under-maintaining and over-maintaining assets. According to [8], “30 to 40% of preventive maintenance costs are spent on assets with negligible failure impact”. Considering this existing situation an optimal maintenance policy is required.

Over time, numerous maintenance policies have been developed, with extensive research dedicated to the subject as indicated by various sources [9,10,11,12,13]. However, the conventional maintenance method of replacing a component only when it fails can prove to be too costly in terms of financial and time resources. An alternative maintenance technique is scheduled preventive maintenance, where preventive replacements are carried out at fixed intervals or when a component reaches a specified age. The optimal replacement interval or the optimal preventive replacement age needs to be determined to optimize this policy [14]. Nonetheless, this policy can significantly raise costs. To minimize costs, it is necessary to consider approaches that reduce over-maintenance, such as condition-based maintenance (CBM). This approach uses condition-monitoring techniques, such as vibration monitoring or thermography, to identify specific defects [15].

This paper is focused on the maintenance cost minimization for the actuators of a proposed harvesting gripper. Section 2 presents the gripper-type harvesting solution using Festo fluidic muscles. The proposed gripper can be mounted on an independent mobile platform that has the capability to detect the crop and harvest it in the fields without human assistance. Due to the lack of a human operator, a certain malfunction can be detected only at the end of the workday when the platform with the gripper returns. Therefore, a preventive maintenance policy is recommended for the gripper actuators in order to minimize the risk of losing a workday and even damaging the crop. The Festo actuators are one of the elements more likely to fail and directly affect the crop and the harvesting process, hence a maintenance cost minimization is conducted for them.

The third part of the paper is focused on briefly presenting the Cuckoo Search (CS) optimization algorithm and the CBM policy used to solve the proposed optimization problem. Section 4 of the paper is devoted to the actual maintenance cost minimization performed in MATLAB and to presenting the obtained results.

## 2. Proposed Harvesting Gripper

**Figure 1:**
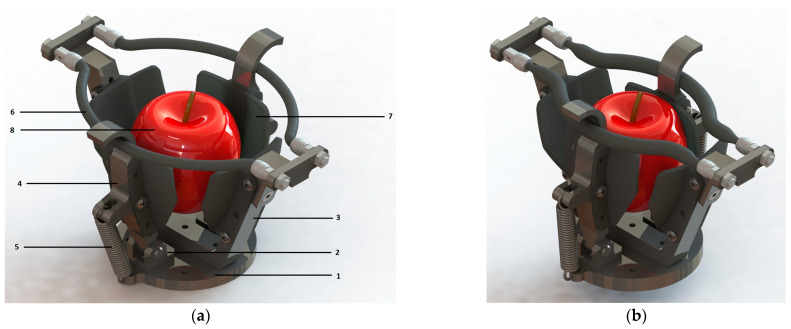
A 3D model for the proposed harvesting gripper in: (**a**) deactivated state, where: 1—base, 2—joint, 3—structural element for sustaining the actuators, 4—mobile connecting element, 5—elastic element (spring) in a compressed state, 6—Festo actuator DMSP-5-150N-RM-CM, 7—rubber part, 8—agricultural product to be harvested; (**b**) activated state.

The proposed harvesting system consists of seven main elements. Its 3D model was designed with SolidWorks 2019 SP03 software, and it is shown in Figure 1a in the deactivated state, and in Figure 1b in the activated state.

The elements are attached to base 1, which is a circular plate with the role of supporting the entire structure. Another role of the base plate 1 is to allow the gripper to be mounted on a manipulator arm by means of which the necessary movements can be made for good positioning in relation to the fruit for a correct harvesting process. Clamping couplings 2 are two rotary couplings clamped to base plate 1. They are mounted diametrically opposite each other by means of two countersunk head screws with an M5 hex socket. Another possibility of mounting them is by welding.

The structural support element 3 is a fixed component mounted along the diameter of base plate 1 so that it is perpendicular to the line joining the 2 rotation couples. The main function of this element 3 is to fix the Festo fluidic muscles, which are attached to its tip with the help of fixing components that are an integrated part of the Festo actuators and the gripper. The structural element supporting the actuators also has another function, namely, that of controlling the gripping direction of the gripper. Due to the particular way of assembly, the Festo fluidic muscles cannot contract in the grip direction, but their activation leads to the approach of the 2 mobile elements 4.

**Figure 2:**
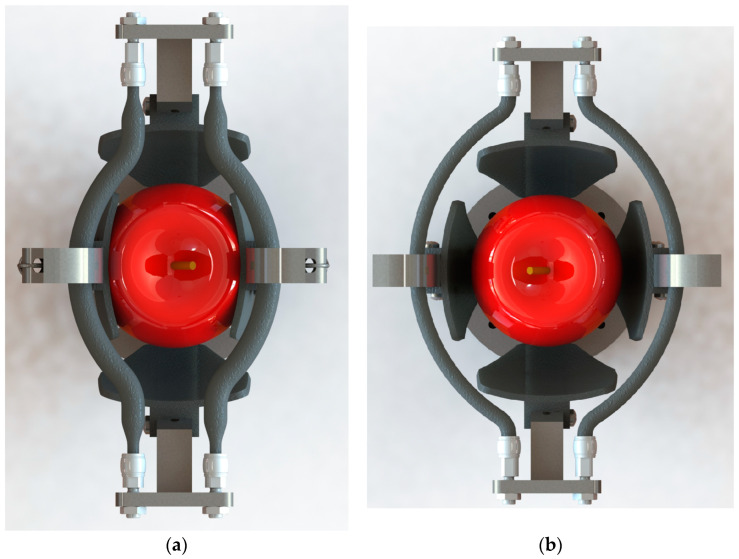
Top view of the 3D model for the proposed harvesting gripper in: (**a**) activated state; (**b**) deactivated state.

The mobile clamping element 4 has a hook-like feature which contains the artificial muscles in the upper part of the gripper and can make a rotational movement thanks to the coupling 2 and the clamping bolt in the plane perpendicular to that of the structural support element 3. Thus, at the moment in which the Festo fluidic muscles are activated, the agricultural product to be harvested is contained according to Figure 1b. Moreover, the hook area, where the muscle directly gets in contact with the mobile connecting element 4, has been designed with a chamfer in order to reduce the sharp edges and prevent the muscle from prematurely bursting. A top view of the 3D model for the proposed gripper is presented in Figure 2 both in activated and deactivated state. The 2 elastic elements 5 are clamped by both the base plate 1 and the mobile clamping elements 4, contributing to the opening of the latter which contain the actuators in the hook-like feature. With the deactivation of the artificial muscles, the elastic elements 5 ensure the opening of the gripper and its preparation in order to grip a new agricultural product.

The last main element of the proposed harvesting system is the 2 Festo fluidic muscles which are actuators activated by air pressure. They tighten the gripper around the agricultural product to be harvested so that the agricultural product 8 can be detached from the stem of the fruit tree (see Figure 1b). To protect the product 8, the gripper has four rubber-like parts 7 which are mounted both on the structural element and on the mobile connecting elements. Because the gripper can be attached to a manipulator arm, it can be programmed to perform complex orienting movements depending on the position of the fruit both for the intended harvest and for directing it to the collection system. The choice of the actuators that have similar operating principle as McKibben artificial muscles, such as the Festo fluidic muscles, is sustained by the McKibben artificial muscles popularity and interest of researchers [16,17]. Of course, McKibben can be used for prototyping and laboratory solutions, whereas Festo offers industrial pneumatic artificial muscles solutions.

Actuation of the artificial muscle and control of the contraction is achieved by adjusting the pressure applied to the muscle. This can be achieved, for example, using a proportional pressure regulator Festo model VPPE-3-1/8-6-010. The proportional pressure regulator controls the pressure in proportion to an analog electrical quantity (voltage or current) reference. The valve operates in a closed loop, the pressure sensor being integrated into the valve.

This pneumatic proportional regulator provides at the output a minimum pressure Pl at the command voltage of 0 V or 4 mA and a maximum pressure Ph at the command voltage of 10 V or 20 mA. The maximum supply pressure is 8 bar and the regulated pressure is 0–6 bar. The supply of the valve is conducted with a continuous voltage of 24 V and the control of the reference is conducted with a voltage of 0–10 V or 4–20 mA.

Because in most harvesting situations a control of the muscle deformation or a limitation of the closing stroke of the gripper is not necessarily desired, the Festo fluidic muscles can be activated by a simple ON/OFF system.

The detection of the actuator state can be performed using a stereo-vision system mounted on the same platform as the gripper, a system also used to detect agricultural products to be harvested. This solution was chosen to reduce the number of electronic components and therefore, to minimize the risk of failure.

More details regarding the structure and operation principle of these types of actuators are described in [18], where the McKibben artificial muscles are considered. In the same paper, the CAD design of a robotic system that incorporates McKibben actuators is included. Moreover, its control system and a developed prototype are also presented in the same paper.

## 3. Materials and Methods

### 3.1. Cuckoo Search Algorithm

**Figure 3:**
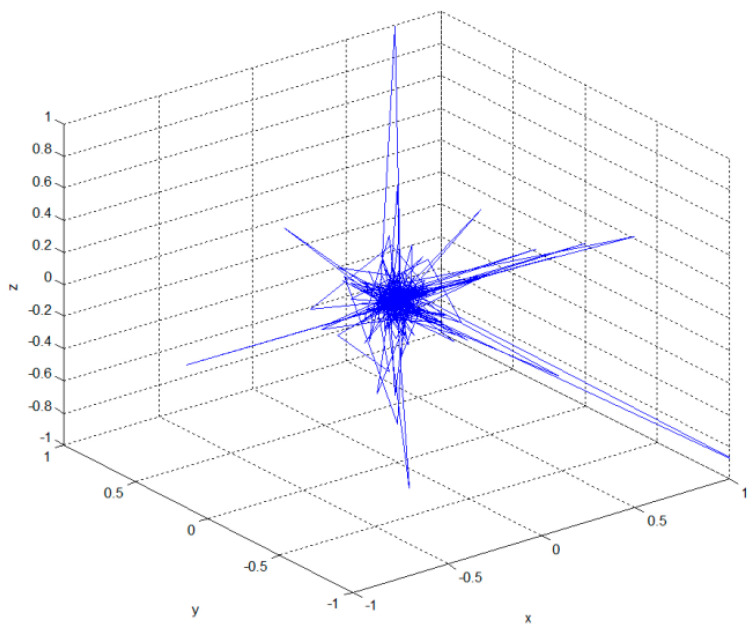
A 3D Lévy flight of 500 steps with (0, 0, 0) as starting point.

The CS optimization algorithm is an evolutionary algorithm inspired by nature, specifically by the breeding behavior of cuckoo birds. These birds are known for being a migratory species that search for areas with more abundant food and better nests for laying their eggs. Interestingly, they do not build their own nests but lay their eggs in communal nests. In 2009, Xin-She Yang and Suash Deb developed the CS algorithm based on this behavior [19]. The migratory flight pattern is based on a type of movement called Lévy flights. Researchers have observed that animals in their natural habitat take a seemingly chaotic path when searching for food, but through the use of probability functions, the search direction can be predicted. Lévy flights are a type of random walk that mimics the search for food of certain animals and insects and have proven successful in natural habitats, leading scientists to incorporate them into some optimization algorithms. A Lévy flight random walk involves a random step length drawn from a Lévy distribution [20].

(1) $$Lévy\;\sim \;u=t^{-\lambda },1<\lambda \leq 3$$

which has infinite mean with an infinite variance. When implementing a few steps, it can be seen that a flight characterized by a batch of small steps is interrupted by significant jumps (Figure 3). A new solution generated based on the random walk using Lévy flights for a given cuckoo *x_i_* is obtained as follows:

(2) $$x_{i}^{\left(k+1\right)}=x_{i}^{\left(k\right)}+\alpha \cdot Lévy(\lambda )$$

where *α* > 0 is the scaling factor of the step size and *k* is the current generation of cuckoos.

The standard version of CS mimicking cuckoo migration using Lévy flights has been customized so that it manages to achieve a balance between exploration and exploitation. A new and improved version of the algorithm was developed starting from the idea that with more knowledge accumulated, not so much exploration is needed. This was achieved by measuring the evolution of knowledge collected by the cuckoo population during migration, measurement made based on the Knowledge Gradient (KG) policy. This improved algorithm was named KGCS [20].

The algorithm was enhanced by introducing two different phases. The first one is dedicated to the search space exploration. In the second one, the intensity of the exploration is diminished while the intensity of the exploitation is boosted.

To achieve maximum exploration of the search space, KGCS uses three populations of cuckoos in its first phase. Initially, the cuckoos within each population are assigned random values within the boundaries of the search space, and their fitness is evaluated based on the value of the objective function. The exploration is carried out independently by all three populations, with more generations obtained by laying eggs in new nests whose positions are determined using Lévy flights. At the end of each generation, the best cuckoo in each population is identified and their values are saved in the corresponding archive. This process continues until a specified stopping criterion is met, ensuring that the algorithm has explored the search space thoroughly.

After 10% of the maximum number of generations has passed, the first phase ends. At this moment, based on the KG policy applied to the existing archives with the best cuckoos, the knowledge gradient value is calculated for each of the three populations. In the next phase, the exploitation is conducted only by the population with the highest knowledge gradient value because, according to the KG policy, it has the largest estimated advancement. The exploitation phase ends when the maximum allowed number of generations is reached. The enhanced KGCS algorithm has been tested on several benchmark functions, as well as used to solve some optimal design problems [20].

### 3.2. CBM Policy

In this subsection, a CBM maintenance policy that was developed and tested in [21] is briefly presented. It was derived from the maintenance policy introduced in [22] where the model does not consider the improvement of the accuracy of predictions. The developed CBM policy is then used to solve a maintenance cost minimization problem for the gripper presented in Section 2, namely when the optimal time to replace the Festo fluidic muscles in order to minimize the maintenance cost of the harvesting system is.

This maintenance policy uses a probability of failure threshold to prevent a premature replacement of a particular component under analysis. More components of the same type are observed over time, inspections are performed at a constant time interval *T*, and data are collected. When conducting an inspection, the estimated probability of failure in the next time interval until the next inspection *Pr_est_* can be calculated. By expressing the total maintenance cost as a function of the probability of failure and performing a minimization of the maintenance cost, the optimal probability of failure threshold *Pr_o_* can be obtained. Based on this optimal threshold, the proposed CBM maintenance policy can be formulated using the following rules [21]:

1. If the component failed during the last inspection interval, a failure replacement will be performed.
2. If the *Pr_est_* value obtained for the next time interval until the next inspection is greater than *Pr_o_*, the decision maker will perform a preventive replacement of the monitored component. Otherwise, the scheduled inspections continue.

**Figure 4:**
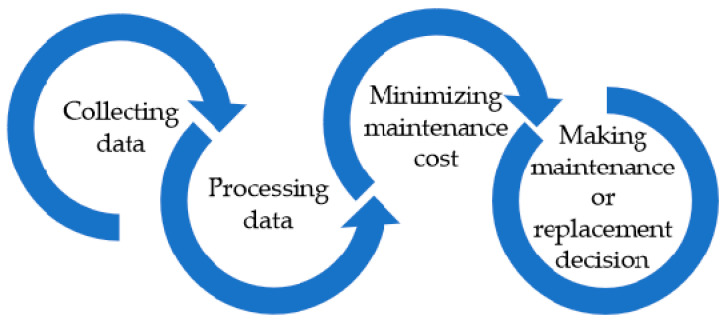
CBM policy’s main steps.

The proposed CBM maintenance policy has four steps (Figure 4). The first step consists in collecting significant data about the operating conditions and lifetime of the monitored components. In the next step, the collected data is processed. If there is no software or mathematical model to predict the lifetime of the monitored component, then an approximative model can be built using some of the collected data, for example using the Kriging interpolation method. The data that are not used to develop the interpolation model can be used to test it. A mean value µ and a standard deviation σ of the lifetime prediction errors is obtained from those tests. Moreover, using the gathered information, a Weibull distribution can be modeled, which is a commonly used life distribution to predict the life characteristics of a component.

The third step is to minimize the maintenance costs. This can be achieved by combining the KGCS optimization algorithm presented in the previous subsection with a stochastic approach in which the expected value is calculated based on small Monte Carlo simulations. In this third step, assuming a normal distribution for the lifetime prediction error, the failure probability estimated in the time interval until the next inspection is computed as follows [21]:

(3) $$Pr_{est}\left(t,t_{P}\right)=\frac{\int_{t}^{t+T}\frac{1}{\sigma \sqrt{2\pi }}e^{-\frac{1}{2}{\left(\frac{x-tp}{\sigma }\right)}^{2}}dx}{\int_{t}^{\infty }\frac{1}{\sigma \sqrt{2\pi }}e^{-\frac{1}{2}{\left(\frac{x-tp}{\sigma }\right)}^{2}}dx}$$

where *t_P_* is the estimated lifetime considering uncertainty and *t* is the current time of inspection.

The predicted failure time with respect to the actual lifetime of the component, *t_W_*, according to the Weibull distribution, is assumed to have a normal distribution, namely *T_pW_~N*(*t_W_*, *σ*^2^). The same assumption is made for the predicted failure time with respect to *t_P_*, namely, *T_pP_~N*(*t_P_*, *σ*^2^), where *t_P_* is computed by subtracting the mean µ from the predicted lifetime.

Given a certain estimated lifetime and a threshold for the failure probability, the preventive replacement time denoted *t_PR_* is determined as the inspection time when the failure probability *Pr_est_* given by Equation (3) becomes greater than the threshold of the failure probability.

Denoting the preventive replacement cost of a component by *C_P_* and the replacement cost in case of failure by *C_F_*, the total expected replacement cost is obtained by computing the sum between the total expected preventive cost *C_TP_* and the total expected replacement cost in case of failure *C_TF_*, where [21]:

(4) $$C_{TP},C_{TF}:[0;1]\times [t_{W}-3\sigma ;t_{W}+3\sigma ]\to (0;\infty )$$

(5) $$C_{TP}(Pr,t_{w})=\sum_{i=1}^{nS}C_{P}\cdot I(t_{PR}(Pr,T_{pP}(i))<t_{w})$$

(6) $$C_{TF}(Pr,t_{w})=\sum_{i=1}^{nS}C_{F}\cdot I(t_{PR}(Pr,T_{pP}(i))\geq t_{w})$$

(7) $$I(t_{PR}(Pr,T_{pP}(i))<t_{w})=\{\begin{array}{cc} 1, & if\;t_{PR}(Pr,T_{pP}(i))<t_{w}\\ 0, & otherwise \end{array}$$

where *nS* is the sample size of the Monte Carlo simulations used to estimate the costs. This sample contains the values *T_pP_*(*i*), *i* = 1,…, *nS* for the predicted failure time taking into account elements of uncertainty, which were generated according to the probability distribution of the random variable *T_pP_*.

Correspondingly, the expected replacement time is obtained by computing the sum between the expected preventive time *T_TP_* and the expected replacement time in case of failure *T_TF_*, where [21]:

(8) $$T_{TP},T_{TF}:[0;1]\times [t_{W}-3\sigma ;t_{W}+3\sigma ]\to (0;\infty )$$

(9) $$T_{TP}(Pr,t_{w})=\sum_{i=1}^{nS}t_{PR}(Pr,T_{pP}(i))\cdot I(t_{PR}\left(Pr,T_{pP}(i)\right)<t_{w})$$

(10) $$T_{TF}(Pr,t_{w})=\sum_{i=1}^{nS}t_{w}\cdot I(t_{PR}\left(Pr,T_{pP}(i)\right)\geq t_{w})$$

Assuming the *α* și *β* are the parameters for the Weibull distribution, the function used to model the total expected cost of the replacement per unit of time with respect to the threshold of the failure probability *Pr* is given by [21]:

(11) $$C_{\exp}:[0;1]\to (0;\infty );C_{\exp}(Pr)=\frac{C_{T}(Pr)}{T_{T}(Pr)}$$

where

(12) $$C_{T},T_{T}:[0;1]\to (0;\infty )$$

(13) $$C_{T}(Pr)=\sum_{j=1}^{nS}\left(C_{TP}(Pr,T_{pW}(j))+C_{TF}(Pr,T_{pW}(j))\right)$$

(14) $$T_{T}(Pr)=\sum_{j=1}^{nS}\left(T_{TP}(Pr,T_{pW}(j))+T_{TF}(Pr,T_{pW}(j))\right)$$

The Monte Carlo simulations used to estimate the expected cost per unit of time have the same sample size *nS*. This time, the sample contains the values *T_pW_*(*j*), *j* = 1,…, *nS* for the predicted failure time with respect to *t_W_*, which were generated according to the probability distribution of the random variable *T_pW_*.

The optimization is carried out with the aim of determining the optimal *Pr_o_* value of the failure probability threshold, a value that minimizes the expected maintenance cost. The optimization problem is a minimization one and can be formulated as follows:

(15) $$\underset{Pr\in [0,1]}{\min}C_{\exp}(Pr)$$

In the fourth and last step of the proposed maintenance policy, the decision to make a preventive replacement is taken only if the calculated failure probability for the next time interval is larger than *Pr_o_*, the optimal probability threshold, and if, of course, the component has not failed in the meantime. A summary of the CBM policy as it was implemented in MATLAB can be found in Table 1.

## 4. Results and Discussion

The problem proposed and solved in this paper consists of determining the best replacement time for the Festo fluidic muscles that minimizes the maintenance cost for the gripper-type harvesting system proposed in Section 2.

For the first step of the CBM maintenance policy, which is to collect historical data on the lifetime of the Festo fluidic muscles, there was no possibility to collect enough data related to the actual lifetime of several such artificial muscles. Therefore, 100 values were randomly generated representing lifetimes for Festo fluidic muscles, which, according to the data provided by the manufacturer Festo, have an average duration of 1,000,000 contractions for a theoretical force of 140 N on the pneumatic muscle at the maximum functioning pressure of 6 bar. The actual mean value of the 100 randomly generated from a Gaussian distribution is 1,001,038.90 contractions, and the standard deviation is 7077.29.

**Figure 5:**
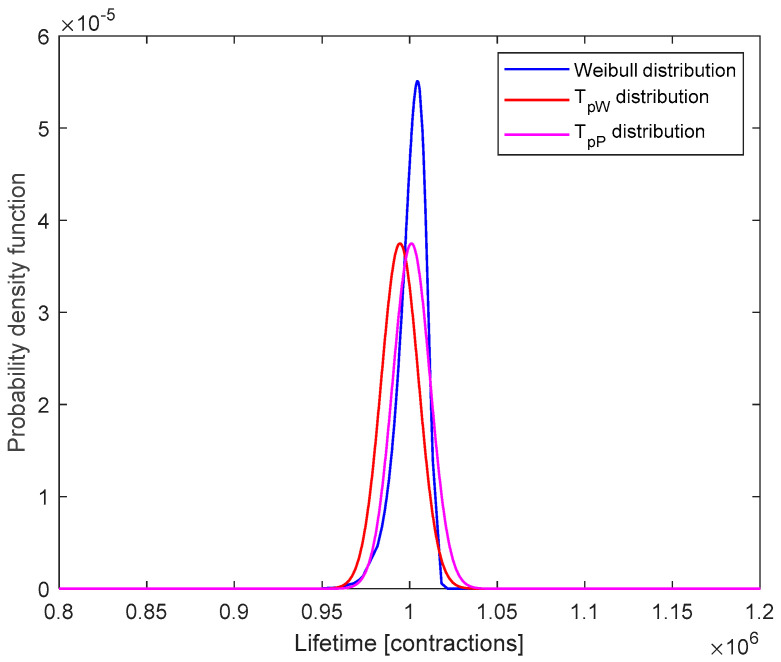
Probability density functions.

In the second step of the proposed CBM policy, the parameters of the Weibull distribution followed by the generated data were determined. The obtained values for the parameters are α = 1,004,527, β = 150.4681, and therefore the actual lifetime of the component according to the Weibull distribution is *t_W_ =* 994,482. Another 100 values were then generated also from a Gaussian distribution to simulate the estimated lifetimes for the Festo fluidic muscles. The average lifetime for the estimated value is 998,443.77 contractions. At this step, the estimated errors were also calculated, and it was determined that they follow the Gaussian distribution with a mean of *µ* = 2595.13 and a standard deviation of *σ* = 10,643.31. Thus, *T_pW_~N*(994,482; 10,643.312) and *T_pP_~N*(100,1038.90; 10,643.312). The graphical representation of the probability density functions corresponding to the Weibull distribution and the distributions of *T_pW_* and *T_pP_* are shown in Figure 5.

In the third step of the CBM maintenance policy, the minimization of the maintenance cost for the proposed harvesting system was carried out using the improved evolutionary algorithm KGCS presented in Section 3.1. The expected cost was estimated using Monte Carlo simulations of 100 values for both the estimated failure time with respect to the actual Festo fluidic muscles lifetime given by the Weibull distribution (*T_pW_*) and the estimated failure time with respect to the duration of their estimated lifetime taking into account elements of uncertainty (*T_pP_*). As a rough estimate, the replacement cost of a set of Festo fluidic muscles is USD 50. If such an actuator fails and the harvesting process doesn’t take place until the failure is discovered at the end of the workday, the total replacement cost is around USD 800 for one gripper, but the total cost is significantly increased when several grippers are attached to different manipulator arms within the same autonomous mobile platform. The optimization was repeated three times using these values and the obtained results are included in Table 2, whereas the statistics regarding these results are included in Table 3. As it can be observed in these tables, the average value for the optimal failure probability threshold is 0.0542 and the approach is stable providing remarkably similar results even if only 100 values were generated for the estimated lifetime of the considered Festo actuators.

**Figure 6:**
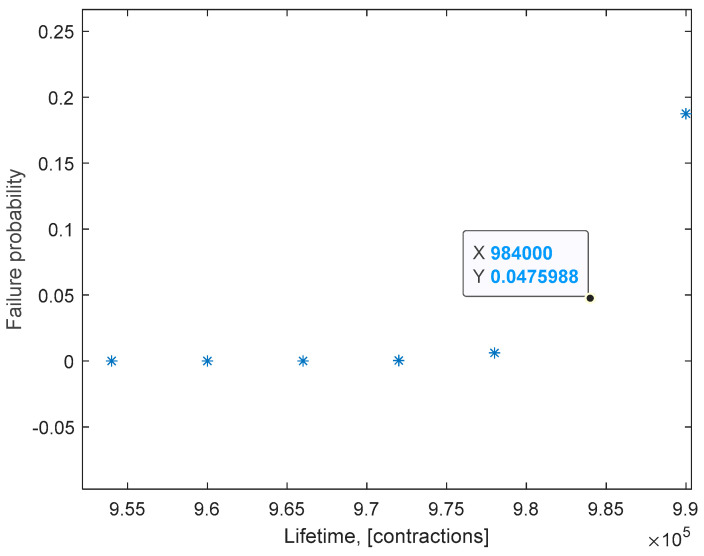
Failure probability at the beginning of each inspection interval.

In the last step, considering the optimal threshold of the probability of failure given by the average of the three results obtained in the optimization step, namely 0.0542, the conditional probability of failure can be determined for each inspection interval. We consider that an inspection takes place at the end of each harvesting day and the Festo fluidic muscles perform daily ~6000 contractions. The values for the conditional failure probability were computed for each inspection interval and the results are illustrated in Figure 6. As it can be observed, the value of the conditional failure probability exceeds the optimal threshold for the interval [984,000; 990,000] contractions. Thus, preventive replacement of the muscles is recommended after 984,000 contractions, a value that corresponds to day 164 of the 166.6 days of operation given by the manufacturer.

For example, if the decision to replace the Festo fluidic muscles is delayed until 990,000 contractions (a value much closer to the value of *t_W_*, according to the Weibull distribution) have been performed, the failure probability increases dramatically and causes a significant increase in the average of the total expected cost per time unit from USD 0.30 per day to USD 5.15 per day. This increase of approximately 17 times when considering the estimated lifetime given by the Weibull distribution becomes quite worrying when not just one gripper is considered but several harvesting platforms with multiple grippers.

## 5. Conclusions

Smart farming is one of the rapidly developing engineering fields because it can substantially cut down the total costs of agricultural production so crucial in a world with high population growth. Harvesting crops is one of the time-consuming and cost-increasing aspects of farming, therefore mechanization and automation are essential, especially for large-scale farming.

The gripper-type harvesting unit proposed in this paper uses Festo fluidic muscles in a novel way, providing a solution easy to manufacture with low maintenance and which can be used for more types of crops due to the actuator choice. Moreover, the gripper can be attached to an independent mobile platform equipped with a vision system that allows crop detection, and therefore, large-scale harvesting can be conducted without human assistance.

The harvesting process can be hindered and the crop can be damaged if one of the Festo fluidic muscles of the proposed harvesting system fails. Hence, an optimal maintenance policy is developed in this paper for the gripper actuators in order to minimize the costs. Due to a lack of available historical data for Festo fluidic muscles, a surrogate model is developed using randomly generated artificial muscle lifetime values in order to illustrate the use and importance of the developed maintenance policy. Using the model and an enhanced evolutive optimization algorithm, the optimal threshold of the failure probability is determined. Based on this threshold, a preventive replacement can be performed in order to minimize maintenance costs.

The results obtained after applying the optimal maintenance policy developed in this paper for the Festo fluidic muscles indicate that a considerable decrement in the costs can be achieved if one performs a preventive replacement of the actuators when the estimated probability of failure in the time interval until the next inspection becomes larger than the determined optimal threshold failure probability. Specifically, a replacement conducted a few days before the lifetime provided by the manufacturer and the lifetime estimated using the well-known Weibull distribution could decrease the maintenance costs approximately 17 times for the Festo fluidic muscles.

The optimal maintenance policy proposed in this paper can be easily customized for other engineering systems and the notable results obtained using this method are proof of its high usefulness in industry.

## Figures and Tables

**Table 1:** Procedure of the proposed CBM policy.

**Step 1**	Collect historical data relevant to the lifetime of the component
**Step 2**	Build the surrogate model based on available historical data Obtain the prediction error based on tests using the surrogate model Determine the distribution of the component lifetime
**Step 3**	Model the expected cost per unit of time in relation to a given failure probability Determine the optimal failure probability, *Pr_o_*, that minimizes the expected cost
**Step 4**	For each inspection point: If the component has failed Make failure replacement Else Calculate failure probability during next inspection interval *Pr_est_* If *Pr_est_* > *Pr_o_* Make preventive replacement End if End if End for

**Table 2:** Results obtained in the maintenance cost optimization.

No.	Optimal Failure Probability	Total Expected Cost per Time Unit [$/day]
1	0.0546	0.3049
2	0.0538	0.3004
3	0.0543	0.3032

**Table 3:** Statistics for the results obtained in the maintenance cost optimization.

Statistics	Optimal Failure Probability	Total Expected Cost per Time Unit [$/day]
Average	0.0542	0.3028
Variance	1.6333 × 10^−7^	5.1633 × 10^−6^
Standard deviation	4.0415 × 10^−4^	0.023

## Data Availability

The data presented in this study are available on request from the corresponding author. The data are not publicly available due to privacy.
